# Non-contiguous finished genome sequence of *Anoxybacillus flavithermus* subsp. *yunnanensis* type strain (E13^T^), a strictly thermophilic and organic solvent-tolerant bacterium

**DOI:** 10.4056/sigs.4968750

**Published:** 2014-03-15

**Authors:** Ying Wang, Yunyun Zheng, Min Wang, Yi Gao, Yazhong Xiao, Hui Peng

**Affiliations:** Anhui Provincial Engineering Technology Research Center of Microorganisms and Biocatalysis, School of Life Sciences, Anhui University, Anhui, P.R. China

**Keywords:** *Anoxybacillus flavithermus* subsp. *yunnanensis*, genome, solvent tolerance, thermophile

## Abstract

*Anoxybacillus flavithermus subsp. yunnanensis* is the only strictly thermophilic bacterium that is able to tolerate a broad range of toxic solvents at its optimal temperature of 55-60°C. The type strain E13^T^ was isolated from water-sediment slurries collected from a hot spring. This study presents the draft genome sequence of *A. flavithermus subsp. yunnanensis* E13^T^ and its annotation. The 2,838,393bp long genome (67 contigs) contains 3,035 protein-coding genes and 85 RNA genes, including 10 rRNA genes, and no plasmids. The genome information has been used to compare with the genomes from *A. flavithermus subsp. flavithermus* strains.

## Introduction

Solvent-tolerant bacteria are a relatively new group of extremophilic microorganisms. They are able to overcome the toxic and destructive effects of organic solvents due to their unique adaptive mechanisms. Most of the reported solvent-tolerant bacteria are mesophilic bacteria that have an optimal temperature of between 25-37°C [1]. So far, *Anoxybacillus flavithermus subsp. yunnanensis* is the only strictly thermophilic bacterial species known to tolerate a broad range of solvents at its optimal temperature of 55-60°C [2,3]. The strains show unusual physiological features in the presence of solvents, such as a higher cell yield [2], an observable incrassation of electron-transparent intracellular material and a distorted cytoplasm [3]. However, mechanisms of solvent tolerance in thermophilic species have not been proposed.

The type strain E13^T^ (=CCTCC AB2010187^T^ =KCTC 13759^T^) and the additional strain PGDY12 were isolated from water-sediment slurries collected from a hot spring in Yunnan Province of China in our lab, and are most closely related to *A. flavithermus* subsp. *flavithermus,* first discovered in a hot spring in New Zealand [4]. At present, a total of 19 species and two subspecies of *Anoxybacillus* with validly published names have been reported [5]. None of these *Anoxybacillus* strains is reported to tolerate solvents except *A. flavithermus subsp. yunnanensis*. To understand the molecular basis of the ability to tolerate solvents under high temperature conditions, we sequenced and annotated a draft genome of the type strain E13^T^ of *A. flavithermus subsp. yunnanensis*.

## Classification and features

*A. flavithermus subsp. yunnanensis* E13^T^ (Table 1) was isolated in 2008 by static cultivation in rich Luria-Bertani (LB) medium supplemented with 10% ethanol [2]. This strain is a facultatively aerobic, Gram-positive, motile, spore-forming rod that is capable of utilizing a wide range of carbon sources, such as arabinose, cellobiose, galactose, maltose, trehalose and xylose. The strain E13^T^ not only exhibited a remarkable ability to grow in ethanol concentrations reaching 13% at 55°C, but can also tolerate highly toxic solvents including toluene, benzene, xylene, chloroform and cyclohexane. Because *A. flavithermus subsp. yunnanensis* is the only strictly thermophilic bacterium that is able to tolerate toxic solvents, the effect of temperature on solvent tolerance has not yet been studied. The reports of the effect of temperature on ethanol (a much less toxic solvent) tolerance indicated that ethanol tolerance decreased with increasing temperature [20,21]. The comparison of the growth of strain E13^T^ at different temperatures showed that a temperature increase of 20°C, from 45 to 65°C, resulted in a decrease of the critical inhibitory toluene concentration from 0.56 to 0.31%. A similar sharp decrease occurred in the cases of benzene, xylene, chloroform and cyclohexane. The results suggested that temperature plays a vitally important role in determining solvent tolerance in bacteria, which may explain why such thermophilic bacteria are rare in nature.

**Table 1:** Classification and general features of *A. flavithermus subsp. yunnanensis* E13^T^ according to the MIGS recommendations [6]

**MIGS ID**	**Property**	**Term**	**Evidence code**^a^
		Domain *Bacteria*	TAS [7]
		Phylum *Firmicutes*	TAS [8-10]
		Class *Bacilli*	TAS [11,12]
	Current classification	Order *Bacillales*	TAS [13,14]
		Family *Bacillaceae*	TAS [13,15]
		Genus *Anoxybacillus*	TAS [16,17]
		Species *Anoxybacillus flavithermus*	TAS [16]
		Subspecies *Anoxybacillus flavithermus subsp. yunnanensis*	TAS [2,18]
		Type strain E13^T^	TAS [2]
	Gram stain	positive	TAS [2]
	Cell shape	rod	TAS [2]
	Motility	motile	TAS [2]
	Sporulation	sporulating	TAS [2]
	Temperature range	30-66°C	TAS [2]
	Optimum temperature	60°C	TAS [2]
	Carbon source	carbohydrates	TAS [2]
	Energy source	heterotrophic	TAS [2]
MIGS-6	Habitat	hot spring	TAS [2]
MIGS-6.3	Salinity	optimum 0.3% (w/v) NaCl	TAS [2]
MIGS-22	Oxygen requirement	facultative anaerobe	TAS [2]
MIGS-15	Biotic relationship	free-living	TAS [2]
MIGS-14	Pathogenicity	non-pathogenic	NAS
MIGS-4	Geographic location	Yunnan, China	TAS [2]
MIGS-5	Sample collection time	2008	IDA
MIGS-4.1	Latitude	N 4°56.5951’	IDA
MIGS-4.2	Longitude	W 98°26.2032’	IDA
MIGS-4.3	Depth	water-sediment slurry (shallow)	IDA
MIGS-4.4	Altitude	1,457 m above sea level	NAS

^a^Evidence codes - IDA: Inferred from Direct Assay (first time in publication); TAS: Traceable Author Statement (i.e., a direct report exists in the literature); NAS: Non-traceable Author Statement (i.e., not directly observed for the living, isolated sample, but based on a generally accepted property for the species, or anecdotal evidence). These evidence codes are from the Gene Ontology project [19]. If the evidence code is IDA, then the property was directly observed by one of the authors or an expert mentioned in the acknowledgements.

**Figure 1:**
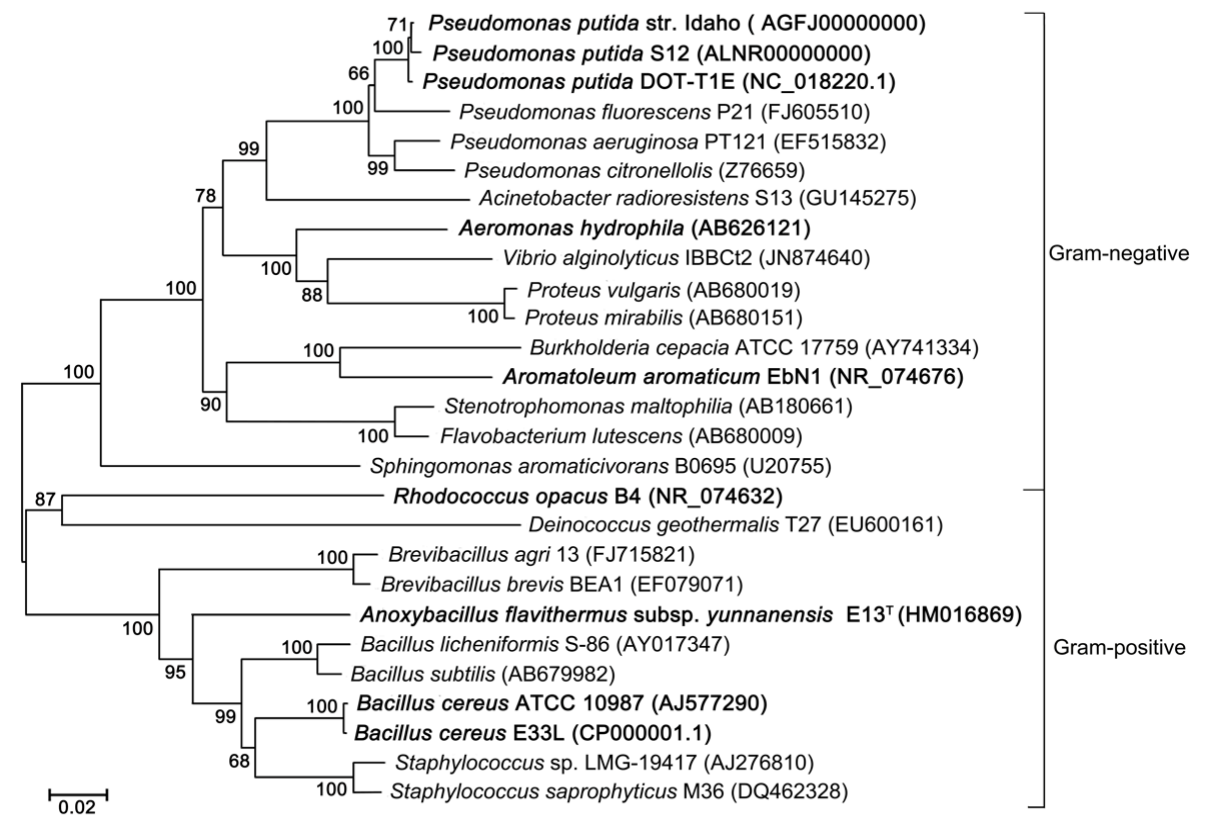
Phylogenetic tree highlighting the position of *A. flavithermus subsp. yunnanensis* E13^T^ relative to other typical solvent-tolerant bacteria. The 16S rRNA sequences were aligned using ClustalX2, and phylogenetic inferences obtained using the neighbor-joining method with the MEGA program. Species and GenBank accession numbers are indicated. Bootstrap values based on 1,000 replicates show the robustness of the branching. Scale bar represents 0.02 substitutions per nucleotide position. Strains with genome sequencing projects registered in GenBank are shown in bold.

Currently, more than 30 solvent-tolerant mesophilic bacteria have been reported, and 8 genomes are available in GenBank. The phylogenetic position of *A. flavithermus subsp. yunnanensis* E13^T^ among these typical solvent-tolerant bacteria is shown in Figure 1. This strain is most closely related to *Bacillus* species. The genomes of *B. cereus* strain E33L and strain ATCC 10987 might provide valuable guidance in a genetic analysis of the solvent tolerance of *A. flavithermus subsp. yunnanensis* E13^T^.

## Genome sequencing information

### Genome project history

The organism was selected based on its unique characteristics as a solvent-tolerant thermophile and in order to investigate new mechanisms of solvent tolerance. The genome was sequenced at BGI-Shenzhen (Shenzhen, China) and deposited in Genbank under the accession number AVGH00000000. The version described in this paper is version AVGH01000000. To our knowledge, it was the first genome of *A. flavithermus subsp. yunnanensis*, the 8th genome of an *Anoxybacillus* species and the 9^th^ genome of solvent-tolerant bacteria to be sequenced. A summary of the project information associated with MIGS version 2.0 compliance [6] is shown in Table 2.

**Table 2:** Project information

**MIGS ID**	**Property**	**Term**
MIGS-31	Finishing quality	High-quality Draft
MIGS-28	Libraries used	One 454 shotgun library and two paired-end Illumina Hiseq 2000 libraries
MIGS-29	Sequencing platforms	454 GS FLX Titanium, Illumina HiSeq 2000 sequencing platform
MIGS-31.2	Fold coverage	52.5×454 Titanium, 368.5×Illumina
MIGS-30	Assemblers	Newbler version 2.6
MIGS-32	Gene calling method	Glimmer 3.02
	Genbank ID	AVGH00000000
	Genbank Date of Release	August 01, 2014
	GOLD ID	Gi0037576
MIGS-13	Project relevance	Strictly thermophilic and organic solvent-tolerant strain

### Growth conditions and DNA isolation

*A. flavithermus subsp. yunnanensis* strain E13^T^ was grown in LB medium at 60°C for 8 h. The cells were harvested by centrifugation at 12,000 g, and washed twice with distilled water. Genomic DNA from the strain E13^T^ was extracted with a Genomic DNA Mini Preparation Kit (Beyotime, Shanghai, China) according to the method for extracting genomic DNA from Gram-positive bacteria. The quality and concentration of the genomic DNA were measured by spectrophotometric analysis using a biophotometer (Eppendorf BioPhotometer Plus, Eppendorf, Germany).

### Genome sequencing and assembly

The genome of *A. flavithermus subsp. yunnanensis* was sequenced using a combination of 454 GS FLX Titanium (Roche) with a shotgun library (1.8-kb insert size), and Illumina Hiseq2000 sequencing platform with two paired-end libraries (0.5 and 6-kb insert size). The 454 shotgun library was constructed with 500 ng of DNA as described by the manufacturer with the GS Rapid library Prep kit (Roche), and the details of Illumina paired-end library construction and sequencing can be found at the Illumina web site. For the genome, we constructed and sequenced a 454 shotgun library which generated 352,901 reads totaling 148.6 Mb, and 2 Illumina paired-end libraries which generated 1,182 Mb raw data. The final assembly was based on 148.6 Mb of 454 draft data, which provides an average 52.5× coverage of the genome and 1,043 Mb of Illumina draft data, which provides an average 368.5× coverage of the genome. These sequences were assembled using Newbler software with 90% identity and 40 bp as overlap. The resulting 67 contigs were scaffolded via read-pairing relationships with SSPACE [22] using all available libraries of high quality reads. The final assembly identified 67 contigs arranged in 24 scaffolds and generated a genome size of 2,838,393 bp.

### Genome annotation

Genes were predicted by merging the results obtained from the RAST (Rapid Annotation using Subsystem Technology) server [23] and the Glimmer modeling software package [24]. The predicted coding sequences (CDSs) were translated and used to search the National Center for Biotechnology Information (NCBI) nonredundant database, KEGG, Clusters of Orthologous Groups (COG), Swiss-Prot and TrEMBL databases. The tool RNAmmer [25] was used to find rRNA genes, whereas tRNA genes were found by using the tool tRNAscanSE [26]. Other non-coding RNAs were identified by searching the genome for Rfam profiles using INFERNAL (v0.81) [27]. Signal peptides and numbers of transmembrane helices were predicted using SignalP [28] and TMHMM [29], respectively.

## Genome properties

**Figure 2:**
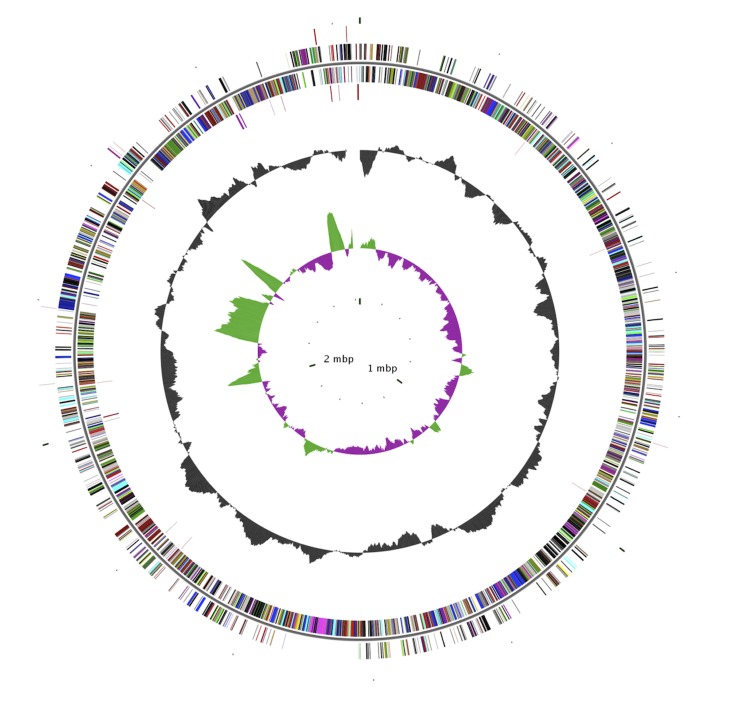
Graphical circular map of the chromosome. From the outside to the center: RNA genes (tRNA red, rRNAs purple and sRNA black) on the forward strand, genes on the forward strand (colored by COG categories), genes on the reverse strand, RNA genes on the reverse strand, G+C content, and GC skew (purple negative values, olive positive values).

The genome is 2,838,393 bp long (1 chromosome, no plasmids) with a 41.4% G+C content (Figure 2 and Table 3). Of the 3,120 predicted genes, 3,035 were protein-coding genes, and 85 were RNAs. In addition, ten rRNA genes (two 16S rRNA, one 23S rRNA and seven 5S rRNA) and 75 predicted tRNA genes were identified in the genome. A total of 2,267 genes (72.66%) were assigned a putative function. The remaining genes were annotated as hypothetical proteins. The properties and the statistics of the genome are summarized in Table 3. The distribution of genes into COGs and KEGG functional categories is presented in Table 4.

**Table 3:** Nucleotide content and gene count levels of the genome

**Attribute**	Value	% of total^a^
Genome size (bp)	2,838,393	100
DNA G+C content (bp)	1,176,230	41.44
DNA Coding region (bp)	2,555,544	90.03
Total genes	3,120	100
RNA genes	85	2.72
Protein-coding genes	3,035	97.28
Genes with protein function prediction	2,267	72.66
Genes assigns to KEGG pathways	1,936	62.05
Genes assigned to KEGG Orthology	1,012	32.43
Genes assigned to COGs	1,886	60.44
Genes with signal peptides	99	3.17
Genes with transmembrane helices	716	22.94

^a^The total is based on either the size of the genome in base pairs or the total number of protein coding genes in the annotated genome

**Table 4:** Number of genes associated with the 25 general COG functional categories

**Code**	**Value**	**%age**^a^	**Description**
J	148	4.88	Translation
A	0	0.00	RNA processing and modification
K	131	4.32	Transcription
L	153	5.04	Replication, recombination and repair
B	1	0.03	Chromatin structure and dynamics
D	27	0.89	Cell cycle control, mitosis and meiosis
Y	0	0.00	Nuclear structure
V	17	0.56	Defense mechanisms
T	93	3.06	Signal transduction mechanisms
M	85	2.80	Cell wall/membrane biogenesis
N	52	1.71	Cell motility
Z	0	0.00	Cytoskeleton
W	0	0.00	Extracellular structures
U	34	1.12	Intracellular trafficking and secretion
O	91	2.99	Posttranslational modification, protein turnover, chaperones
C	137	4.51	Energy production and conversion
G	144	4.74	Carbohydrate transport and metabolism
E	200	6.59	Amino acid transport and metabolism
F	63	2.08	Nucleotide transport and metabolism
H	103	3.39	Coenzyme transport and metabolism
I	79	2.60	Lipid transport and metabolism
P	122	4.02	Inorganic ion transport and metabolism
Q	32	1.05	Secondary metabolites biosynthesis, transport and catabolism
R	231	7.61	General function prediction only
S	169	5.57	Function unknown
-	1,234	40.66	Not in COGs

a) The total is based on the total number of protein coding genes in the annotated genome.

## Comparison with other *Anoxybacillus flavithermus* genomes

As of this moment, six genome sequences from *Anoxybacillus* species are available in GenBank database, including four *A. flavithermus subsp. flavithermus* strains, one *A. kamchatkensis* strain and one *Anoxybacillus* sp. strain. Only *A. flavithermus subsp. flavithermus* strain WK1 and strain TNO-09.006 have complete genome sequences [30,31]. Here we compare the genome sequence of *A. flavithermus subsp. yunnanensis* E13^T^ with those of the four *A. flavithermus subsp. flavithermus* strains. The draft genome of *A. flavithermus subsp. yunnanensis* E13^T^ is similar in size to that of *A. flavithermus subsp. flavithermus* strain WK1 (2.83 vs 2.84 Mb, respectively), but larger than that of strain TNO-09.006, strain AK1 and strain NBRC 109594 (2.65, 2.63 and 2.77 Mb, respectively). The G+C content of *A. flavithermus subsp. yunnanensis* E13^T^ is similar to those of *A. flavithermus* subsp. *flavithermus* strain WK1, strain TNO-09.006 and strain NBRC 109594 (41.4, 41.7, 41.8 and 41.7%, respectively), but slightly less than that of strain AK1 (42.7%). The gene content of *A. flavithermus subsp. yunnanensis* E13^T^ is greater than those of *A. flavithermus subsp. flavithermus* strain WK1, strain TNO-09.006, strain AK1 and strain NBRC 109594 (3,120, 2,954, 2,819, 2,799 and 2,963 genes, respectively). In addition, *A. flavithermus subsp. yunnanensis* E13^T^ shared a mean genome sequence similarity of 90% (range 80-99%), 90% (79-100%), 86% (73-99%) and 91% (71-100%) with *A. flavithermus subsp. flavithermus* strain WK1, strain TNO-09.006, strain AK1 and strain NBRC 109594, respectively.
